# Towards Sustainable Environmental Quality: Priority Research Questions for the Australasian Region of Oceania

**DOI:** 10.1002/ieam.4180

**Published:** 2019-09-13

**Authors:** Sally Gaw, Andrew Harford, Vincent Pettigrove, Graham Sevicke‐Jones, Therese Manning, James Ataria, Tom Cresswell, Katherine A Dafforn, Frederic DL Leusch, Bradley Moggridge, Marcus Cameron, John Chapman, Gary Coates, Anne Colville, Claire Death, Kimberly Hageman, Kathryn Hassell, Molly Hoak, Jennifer Gadd, Dianne F Jolley, Ali Karami, Konstantinos Kotzakoulakis, Richard Lim, Nicole McRae, Leon Metzeling, Thomas Mooney, Jackie Myers, Andrew Pearson, Minna Saaristo, Dave Sharley, Julia Stuthe, Oliver Sutherland, Oliver Thomas, Louis Tremblay, Waitangi Wood, Alistair BA Boxall, Murray A Rudd, Bryan W Brooks

**Affiliations:** ^1^ School of Physical and Chemical Sciences University of Canterbury Christchurch New Zealand; ^2^ Department of the Environment and Energy Australian Government, Darwin Australia; ^3^ Aquatic Environmental Stress Research Centre RMIT University, Bundoora Victoria Australia; ^4^ Environment Southland Regional Council Invercargill New Zealand; ^5^ Environmental Risk Sciences Sydney Australia; ^6^ Cawthron Institute Nelson New Zealand; ^7^ Australia's Nuclear Science and Technology Organisation Lucas Heights Australia; ^8^ Department of Environmental Sciences Macquarie University North Ryde Australia; ^9^ Australian Rivers Institute and School of Environment and Science Griffith University Brisbane Australia; ^10^ Institute for Applied Ecology University of Canberra Canberra Australia; ^11^ Auckland Council Auckland New Zealand; ^12^ Office of Environment and Heritage New South Wales Australia; ^13^ Te Rūnanga o Ngāi Tahu Christchurch New Zealand; ^14^ School of Life Sciences University of Technology Sydney Sydney Australia; ^15^ Faculty of Veterinary Science University of Melbourne Victoria Australia; ^16^ Department of Chemistry and Biochemistry Utah State University, Logan Utah USA; ^17^ School of Biosciences The University of Melbourne, Parkville Victoria Australia; ^18^ National Institute of Atmospheric and Water Research Auckland New Zealand; ^19^ Faculty of Science, University of Technology Sydney Sydney Australia; ^20^ Environmental Futures Research Institute Griffith University Brisbane Australia; ^21^ Environment Protection Authority Victoria Australia; ^22^ Macquarie University Sydney Australia; ^23^ Ministry for Primary Industries Wellington New Zealand; ^24^ School of Biological Sciences Monash University Melbourne Australia; ^25^ Bio2Lab, Melbourne Innovation Centre Greensborough Australia; ^26^ CSIRO Publishing, Clayton Victoria Australia; ^27^ Independent researcher Nelson New Zealand; ^28^ School of Applied Chemistry and Environmental Science RMIT University, Melbourne Victoria Australia; ^29^ School of Biological Sciences University of Auckland Auckland New Zealand; ^30^ Tau Iho I Te Po Trust Kaeo New Zealand; ^31^ Environment Department University of York York United Kingdom; ^32^ World Maritime University Malmö Sweden; ^33^ Baylor University Waco Texas USA

**Keywords:** Multiple stressors and mixtures, Risk assessment, Contaminants of emerging concern, Indigenous knowledge, Cultural values

## Abstract

Environmental challenges persist across the world, including the Australasian region of Oceania, where biodiversity hotspots and unique ecosystems such as the Great Barrier Reef are common. These systems are routinely affected by multiple stressors from anthropogenic activities, and increasingly influenced by global megatrends (e.g., the food–energy–water nexus, demographic transitions to cities) and climate change. Here we report priority research questions from the Global Horizon Scanning Project, which aimed to identify, prioritize, and advance environmental quality research needs from an Australasian perspective, within a global context. We employed a transparent and inclusive process of soliciting key questions from Australasian members of the Society of Environmental Toxicology and Chemistry. Following submission of 78 questions, 20 priority research questions were identified during an expert workshop in Nelson, New Zealand. These research questions covered a range of issues of global relevance, including research needed to more closely integrate ecotoxicology and ecology for the protection of ecosystems, increase flexibility for prioritizing chemical substances currently in commerce, understand the impacts of complex mixtures and multiple stressors, and define environmental quality and ecosystem integrity of temporary waters. Some questions have specific relevance to Australasia, particularly the uncertainties associated with using toxicity data from exotic species to protect unique indigenous species. Several related priority questions deal with the theme of how widely international ecotoxicological data and databases can be applied to regional ecosystems. Other timely questions, which focus on improving predictive chemistry and toxicology tools and techniques, will be important to answer several of the priority questions identified here. Another important question raised was how to protect local cultural and social values and maintain indigenous engagement during problem formulation and identification of ecosystem protection goals. Addressing these questions will be challenging, but doing so promises to advance environmental sustainability in Oceania and globally.

## Key Points

- The first process to identify priority research questions for the Australasian region of Oceania.
- Priority areas identified included integration of ecology and ecotoxicology, impacts of multiple stressors, and using data from exotic species to protect indigenous species.
- The need to protect local cultural and social values and maintain indigenous peoples’ engagement during problem formulation and identification of ecosystem protection goals was also highlighted.

## INTRODUCTION

Achieving sustainable environmental quality and ecosystem integrity is a critical goal shared by diverse stakeholders around the world. Unimpaired and diverse ecosystems conserve biodiversity and provide essential ecosystem services, while being more resilient when natural and anthropogenic disasters occur (Alexander et al. 2016). The United Nations Sustainable Development Goals aim to protect the planet and realize prosperity for all people, including future generations (UN 2015). Within this framework are interconnected goals that inherently rely on achieving more sustainable environmental quality and ecosystem integrity. But achieving these goals depends on effective environmental management efforts informed by the best available scientific knowledge and technological advancements. Integration of robust environmental risk assessment with ecosystem protection goals is therefore critical in light of global megatrends (e.g., the food–energy–water nexus, demographic transitions to cities) and climate change that present unique challenges for policy makers and environmental and health professionals. These sustainable management challenges are complex, particularly given environmental, political, and economic contexts that exist among and within global regions.

**Figure 1:**
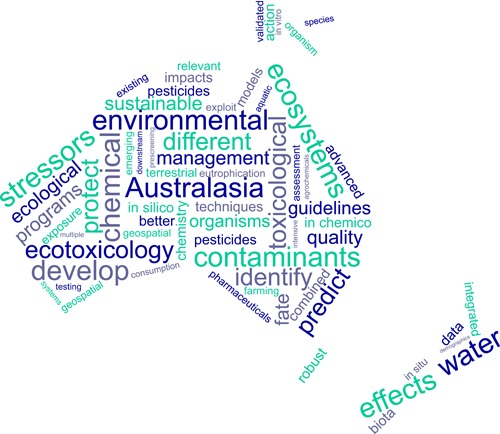
Word cloud of priority research questions from the Australasian Region of Oceania.

Intersections of biodiversity, environmental variability, and anthropogenic stressors are pronounced in the Australasian region of Oceania. Countries in the region have iconic landscapes with unique flora and fauna. The ability to participate in outdoor activities, including hiking, camping, fishing, and swimming, is treasured in Australia and New Zealand and considered to be part of their national identities (Garner 2013; McCrone 2017). Biodiversity hotspots are prevalent, as are freshwater and marine ecosystems (e.g., the Great Barrier Reef), which are susceptible to stress from anthropogenic activities, including climate change (Adams et al. 2016). Interconnections among stressors from landscape development and urbanization across freshwater to marine gradients are widespread in this region (e.g., Mayer‐Pinto et al. 2015; Weeks et al. 2016), where the vast majority of human populations reside within 50 km from the coast.

Climate change is significantly affecting the island nations of Oceania (Caritas 2018) and magnifying the importance of understanding how multiple physical and chemical stressors impact biodiversity and ecosystem services (Weeks et al. 2016). However, information on the influences of natural and anthropogenic stressors, particularly chemical contaminants, is scarce for native species. There are also relatively few ecotoxicology data sets of relevance to the tropical conditions of Papua New Guinea and much of the northern parts of Australia. Degradation of water quality is of particular concern for Māori and Aboriginal communities. There is growing appreciation of the spiritual and cultural values and developmental aspirations of indigenous communities; momentum is building to incorporate these in environmental policies and decision making (Bark et al. 2015; Harmsworth et al. 2016; Ataria et al. 2018). Unfortunately, identifying global priority environmental quality research needs to attain these ecosystem protection goals and effectively implement policy instruments has remained elusive on a regional scale. Horizon‐scanning approaches for identifying key research questions may be part of developing sustainable solutions.

Horizon scanning using a key questions approach has emerged from the conservation sciences, public health, and other disciplines as an effective means to identify important research needs through engagement of diverse stakeholders (Sutherland and Woodroof 2009; Boxall et al. 2012; Rudd et al. 2018). The Global Horizon Scanning Project (GHSP) was initiated with the Society of Environmental Toxicology and Chemistry (SETAC) to identify priority research questions that advance understanding of how environmental stressors impact environmental quality (Brooks et al. 2013). This initiative is collecting and prioritizing the most important current and emerging research questions related to environmental quality as recognized by scientists and engineers from multiple disciplines working in government, academia, and business around the globe. For example, priority research questions were recently reported from Latin America (Furley et al. 2018), Europe (Van den Brink et al. 2018), and North America (Fairbrother et al. 2019). Here we specifically present results from the GHSP project focused on the Australasian region of Oceania. The scope of these questions was intended to be of relevance to Australasia, within a global context. We anticipate these priority research questions will be indispensable in informing and structuring research agendas by the government and business communities in the future.

## METHODS

In the present study, we followed previously reported methods (Boxall et al. 2012; Furley et al. 2018; Van den Brink et al. 2018) to identify priority research questions. Prior to holding a workshop in Nelson, New Zealand, in 2015, members of SETAC and other scientists from Oceania were asked to submit research questions which, in their view, were priority environmental quality research needs to address. Consistent with methods employed in other studies (Sutherland et al. 2011) and SETAC geographic regions (Furley et al. 2018; Van den Brink et al. 2018), participants were provided criteria for an ideal question, which should address important gaps in knowledge; be answerable through a realistic research design; have a factual answer that does not depend on value judgments; cover a spatial and temporal scale that could realistically be addressed by a research team; not be answerable by “it all depends,” “yes,” or “no”; and if related to impact and interventions, the research question should contain a subject, an intervention, and a measurable outcome. In total, 78 questions were received and are presented in Supplemental Data.

Before the workshop, questions were partitioned among 6 themes, including contaminants of emerging concern; environmental chemistry: analysis, fate, and exposure; multiple stressors and mixtures; risk assessment, regulations, and guidelines; spotlight on Australasia; and tools for improving risk assessment. These 6 themes were used to structure an expert workshop held in Nelson, New Zealand as part of the SETAC Australasia meeting in 2015 at which the questions were discussed. During the workshop, 20 priority research questions were identified by participants from academic, business, the indigenous community, and government sectors. We specifically examine each of these priority research questions in the sections that follow (Table 1).

**Table 1:** Top 20 priority research questions from the Australasian portion of the Global Horizon Scanning Project by theme

Themes and priority research questions
**Contaminants of emerging concern**
What are the most appropriate toxicological approaches to develop regulatory guidelines specifically for contaminants of emerging concern that address multimodes of action and sublethal effects?
How can we identify and prioritize contaminants (traditional and emerging stressors) for sustainable management of ecosystems within different biogeographic regions?
How can we identify and examine the environmental fate and toxicity of ingredients other than the stated “active” components in commercial formulations, individually and in chemical mixtures?
**Environmental chemistry: Analysis, fate, and exposure**
How can we develop robust chemical assays and models to replace, refine, and reduce biological testing?
How do we better understand the linkages between the structural and physicochemical properties of substances to predictively model fate and bioavailability in different environments?
How do we develop better broad‐screening analytical and information‐processing techniques that do not require preselection of target contaminants?
How do we use chemistry to better design sustainable waste management?
How can we ensure sustainable supplies of clean water, energy development, and food security while simultaneously minimizing ecological impacts and protecting environmental quality?
**Multiple stressors and mixtures**
What are the combined impacts of various agrochemicals (e.g., veterinary medicines, pesticides) and eutrophication from intensive terrestrial farming operations on the health of aquatic and terrestrial organisms?
What are the effects of changing demographics, economic development, consumption patterns, and climate (e.g., ocean acidity, water temperature) on chemical emissions, environmental fate, and ecotoxicology of contaminants and multiple stressors?
What are the combined effects of very low levels of multiple contaminants (e.g., pesticides, natural resource extraction contaminants, salinity, pharmaceuticals and personal care products, endocrine‐disrupting chemicals) with different modes of action on aquatic and terrestrial organisms and ecosystems?
**Risk assessment, regulations and guidelines**
What water quality guidelines are needed to protect temporary waters and associated ecosystems from the influences of development?
What are the effects of short magnitude, frequency, and duration (e.g., intermittent, episodic) exposures to contaminants and other stressors, and how can these scenarios be effectively incorporated into water quality guidelines?
How can we measure ecosystem resilience to and recovery following exposure to stressors?
**Spotlight on Australasia**
Are there differences in toxicological thresholds among native and nonnative organisms, and how can species sensitivity information from nonresident species be used to predict adverse outcomes and protect our unique biota and ecosystems?
How do we incorporate and protect cultural and social values (relating to humans, biota, and ecosystems) to empower citizen, societal, and indigenous engagement in the research, management, and legislation of priority environmental contaminants?
**Tools for improving risk assessment**
How do we exploit, collate, and integrate existing environmental toxicology, chemistry, and geospatial data to help develop robust risk assessment?
How can prescreening techniques (e.g.*,* in silico, in vitro) be developed, advanced, and validated to identify and predict whole organism effects?
How can ecotoxicology information be integrated more closely during interpretation of ecological data?
How do we advance ecotoxicology testing to be more relevant to ecological systems?

## CONTAMINANTS OF EMERGING CONCERN

### What are the most appropriate toxicological approaches to develop regulatory guidelines specifically for contaminants of emerging concern (CECs) that address multimodes of action and sublethal effects?

Measures of effect are selected during problem formulation in ecological risk assessment to support assessment endpoints that are aligned with ecosystem protection goals (Suter 2006). Historically, these measures of effect include a limited number of model organisms and endpoints (survival, growth, reproduction) linked to adverse outcomes of importance to the population level and environmental management. Single‐species ecotoxicity information for a specific chemical is then routinely utilized to develop species sensitivity distributions from which water quality criteria, standards, or guidelines are derived around the world (Posthuma et al. 2001). Recent revisions of Australia and New Zealand Environment and Conservation Council (ANZECC, now referred to as the Australian and New Zealand Governments [ANZG] 2018) guidelines, and the use of multiple lines of evidence in weight‐of‐evidence assessments, represent global steps forward consistent with global trends.

Although recent years have seen an increase in the use of chronic toxicity testing with Australasian species, a historical overreliance on a limited number of model organisms and endpoints has potentially undermined management activities related to sustainable environmental quality and ecosystem integrity. Much of the available ecotoxicology information has been primarily comprised of acute lethality responses of several species (e.g., *Daphnia* sp.) from the Northern Hemisphere. Sublethal responses to chemical stressors were primarily available for cladoceran reproduction, microalgal growth rate, and juvenile fish growth. Similar model organisms and endpoints also have been employed for whole effluent (aka “direct toxicity assessments”) and ambient toxicity testing (USEPA 1991). However, assays based on these model organisms and endpoints were often not developed to account for mutagenicity, teratogenicity, and other adverse outcomes that result from diverse molecular initiation events (MIEs; Ankley et al. 2010). Other ecologically important endpoints, including developmental and behavioral responses, are increasingly receiving attention in terms of potential importance (e.g., Saaristo et al. 2018).

Early research with endocrine‐disrupting and ‐modulating chemicals (EDCs) recognized some of the limitations of these traditional tools to assess environmental quality and to derive guideline values protective of aquatic systems. For example, a 6‐order‐of‐magnitude difference exists between adverse effects on cladoceran (Clubbs and Brooks 2007) versus fish reproduction (Kidd et al. 2007) elicited by the human estrogen agonist 17α‐ethinylestradiol because invertebrates do not possess a functional estrogen receptor (Ankley et al. 2016). After almost a decade of health and ecological research on EDCs, Ankley et al. (2007) identified that such lessons learned from these chemicals were important to understanding risks of pharmaceuticals in the environment. Subsequently, efforts such as the development of adverse outcome pathways (AOPs; Ankley et al. 2010), informed by comparative pharmacology and toxicology research (LaLone et al. 2016; Brooks 2018), have been advancing the use of pathway‐based predictive approaches in ecological risk assessment.

In parallel, buoyed by release of Toxicity Testing in the 21st Century (NRC 2007), the Tox21 and ToxCast programs were launched (Dix et al. 2006). These have screened thousands of chemicals with hundreds of in vitro assays, largely adapted from drug discovery and safety testing programs, to identify likely MIEs associated with many untested chemicals. These and related next‐generation risk assessment efforts are breaking new ground (Cote et al. 2016). For example, identification of diverse MIEs associated with chemical properties is supporting development of next‐generation computational toxicology models to identify problematic (and useful) substances and to sustainably design less hazardous chemicals. More recent applications include employing these in vitro systems for prioritizing environmental assessments (Li et al. 2017) and performing cross‐species extrapolation (LaLone et al. 2018), or tracking movements of multiple individuals simultaneously using ToxTrac (Rodriguez et al. 2018). Such efforts promise to continue to further advance environmental risk assessment practices (Villeneuve et al. 2019).

Integrating comparative toxicology information and mechanistic tools such as high‐throughput assays with regulatory guideline development and environmental monitoring and assessment represents important research needs. In the case of pharmaceuticals, for example, short‐term standardized ecotoxicity test model species and endpoints are often not adequate to define chronic toxicity (Brooks 2018). Herein, therapeutic hazard values (Brooks 2014) and minimal selective concentrations and associated predicted no‐effect concentrations for the development of antibiotic resistance by microorganisms in particular (Bengtsson‐Palme and Larsson 2016) represent recent approaches to identify water concentrations supporting more robust ecological and human health water quality assessments, respectively, and to further support environmental diagnostic applications. However, integrative, comparative, and predictive toxicology research must be advanced to understand ecologically important effects caused by new and poorly studied chemicals.

### How can we identify and prioritize contaminants (traditional and emerging stressors) for sustainable management of ecosystems within different biogeographic regions?

Like other regions of the globe, Australasian ecosystems are subject to a variety of chemical and other stressors, which challenges stressor identification research and practice. However, due to the smaller economies of the Australasian region, it is especially not feasible to have ever‐expanding monitoring lists for contaminants, and care is needed to avoid needless selection of priority contaminants based on data from different biogeographic regions of Australasia. Risk‐based frameworks for identification and prioritization of contaminants that incorporate local ecosystem‐specific vulnerability to contaminants and Australasia‐specific use of chemicals are urgently needed. Factors contributing to Australasia‐specific use of chemicals include regulatory decisions, patents, demographics, land use, and climate, along with human and animal disease and pest profiles (Daughton 2014; Kookana et al. 2014; Gaw and Brooks 2016). These factors will change over time, and prioritization schemes and ultimately regulatory and monitoring regimes will need to be sufficiently agile and adaptive to examine substances currently in commercial use. Solid waste and wastewater management practices in Australasia will also determine priority substances in the region. In addition to anthropogenic chemical contaminants, transformation products and endogenous biomolecules, including toxins from harmful algal blooms (HABs), need to be assessed. Other important stressors that also need to be taken into consideration include changing land use, urbanization, climate change, and biological stressors such as predation, overexploitation, and invasive species. Globally, the need for contaminant prioritization has been identified for pharmaceuticals and personal care products (Boxall et al. 2012), microplastics (Eerkes‐Medrano et al. 2015), and pesticides and their transformation products (Sinclair et al. 2006). Ultimately, risk‐based identification and prioritization frameworks for contaminants, which are currently used by Australasian chemical management authorities, need to be diligently updated to reflect contemporary uses and potential exposure. They also need to be further developed to be broader than single classes of contaminants and to incorporate nonchemical stressors.

### How can we identify and examine the environmental fate and toxicity of ingredients other than the stated “active” components in commercial formulations, individually and in chemical mixtures?

Ecotoxicity testing is generally focused on known active components as pure substances rather than as components of commercial formulations and chemical mixtures. Many products contain ingredients other than the stated active components to enhance the stability or performance of the product. Examples include adjuvants added to pesticides, coloring agents and preservatives added to soaps, fragrances added to cleaning products, and a wide range of excipients added to pharmaceutical products. These “other” or “inert” ingredients have the potential to alter the environmental fate and toxicity of the active components in commercial formulations as well as in other contaminants and may also present their own inherent hazards and risks (Cox and Surgan 2006). For example, glyphosate formulations containing surfactants were more toxic than glyphosate on its own (Vincent and Davidson 2015). Such ingredients may not be listed, especially for proprietary formulations, making it difficult to identify and prioritize components of formulations for study. Identification of potentially problematic ingredients in products other than the active ingredient will lead to improved risk assessment and ultimately to safer products.

## ENVIRONMENTAL CHEMISTRY: ANALYSIS, FATE, AND EXPOSURE

### How can we develop robust chemical assays and models to replace, refine, and reduce biological testing?

Globally there is a focus on reducing biological testing to reduce the numbers of animals used in testing and to minimize the costs and time involved (e.g., Hutchinson et al. 2016). Additionally, the ever‐increasing volume and classes of chemicals in widespread use makes comprehensive biological testing unfeasible. Consequently, in silico toxicology efforts that commonly employ quantitative structure–activity relationships (QSARs) have become critical for early tier assessments of industrial chemicals (Myatt et al. 2018). The AOP approach has been proposed as a tool to help assess the safety of chemicals that, when coupled with robust computational toxicology, will reduce reliance on biological testing (Burden et al. 2015). Importantly, more research efforts should be targeted at predictively identifying chemical properties that result in MIEs with adverse outcomes at the organism and population levels. Also as noted above, one such attempt is the United States Environmental Protection Agency's (USEPA) ToxCast program, which employs computational and high‐throughput screening (HTS) tools for prioritizing environmental contaminants (Dix et al. 2006; Cote et al. 2016). In fact, molecular docking (McRobb et al. 2014) and quantum mechanics approaches are advancing the science beyond traditional log *K*_ow_ based QSAR approaches (Kostal 2018).

### How do we better understand the linkages between the structural and physicochemical properties of substances to predictively model fate and bioavailability in different environments?

Structural and physicochemical properties of compounds are used in risk assessments to identify priority persistent and bioaccumulative compounds (Howard and Muir 2010). Many of the algorithms used in risk assessments were developed for hydrophobic organic compounds under temperate conditions. There is increasing evidence that these “rules of thumb” developed for neutral hydrophobic compounds may not be sufficiently predictive of the fate and bioavailability of hydrophilic compounds and do not predict the behavior of ionizable compounds. For example, the octanol–water partition coefficient log *K*_ow_ is used as an indicator of enhanced accumulation, with molecules that have log *K*_ow_ values greater than 3 predicted to accumulate. However, some uncharged molecules with low log *K*_ow_ values have also been shown to accumulate in organisms (e.g., Emnet et al. 2015). Similarly, *K*_ow_‐based approaches have limitations for ionizable chemicals such as pharmaceuticals and per‐ and polyfluoralkyl substances (PFAS), which partition by nonhydrophobic mechanisms (e.g., ion exchange, protein binding; Armitage et al. 2017). There is a need to undertake a metaanalysis of the available data on the linkages between the structural and physicochemical properties of substances and their environmental fate and bioavailability. Basic and applied research will be necessary to improve predictive models for properties that fall outside of the mechanistic domain of historic hydrophobic contaminants.

### How do we develop better broad‐screening analytical and information‐processing techniques that do not require preselection of target contaminants?

“You only find what you are looking for” is a truism of environmental monitoring (Waller and Allen 2008). Widely available analytical techniques require preselection of target analytes and commonly include extensive sample preparation. This approach means that environmental monitoring programs selectively include known contaminants for which robust analytical methods exist and may not provide data on the priority contaminants for a particular time or location (Daughton 2014; Gaw and Brooks 2016). Analysis costs associated with screening just 1 water sample, for example, can be prohibitive when using multiple traditionally available analytical methods for diverse classes of contaminants. In addition, it can be difficult to establish whether there are no data for a particular contaminant because it is not present in the environment or because there are no suitable analytical methods and standards. Although new approaches using high‐resolution mass spectrometry are being developed to enable nontarget analysis of organic compounds (Samanipour et al. 2016; Hollender et al. 2017), these techniques are not yet routine and provide information only on organic classes of contaminants. In contrast, ecosystems are exposed to complex mixtures that contain nutrients and metals, in addition to synthetic and naturally produced organic compounds. Advancing development and availability of robust nontarget screening techniques would significantly enhance environmental protection and would specifically support a number of the other top 20 research questions identified here.

### How do we use chemistry to better design sustainable waste management?

Global pollution is now recognized as being responsible for the loss of more human lives each year than all wars or cancers (Landrigan et al. 2018). Human population growth and urbanization results in product use and chemical consumption being concentrated in cities faster than environmental management systems and interventions are being developed (Brooks 2018). For example, solid waste generation, which is currently estimated at 10 billion tons per year in urban areas, will continue to grow and become increasingly concentrated, particularly in developing and middle‐income countries (Wilson et al. 2015). In Australia, although per capita waste generation has decreased, the mass of solid waste produced continues to increase, with a 7 % increase over a recent 11‐y period (over the period of 2006–2007 to 2016–2017; National Waste Report 2018). New Zealand is one of the highest generators of household waste in the Organisation for Economic Co‐operation and Development (OECD 2019). Similarly, wastewater production is concentrated in cities, yet 80 % of the global sewage production is released untreated to the environment (WWAP 2017). Key sustainable development goals aim to increase sustainable cities and communities as well as responsible consumption (UN 2015), which will require development and implementation of innovative waste management programs. Advancing green engineering to reduce waste generation, increasing beneficial reuse and recovery from diverse waste streams, and stimulating sustainable molecular design of chemical ingredients and products that maintain function but are less hazardous and degrade faster (Coish et al. 2016) represent important opportunities to meet sustainability goals while stimulating innovation and reducing chemical risks to public health and the environment. In fact, designing a future without waste and associated environmental pollution was recently identified as a grand challenge for environmental engineering (NASEM 2018). To realize this challenge, environmental toxicology, chemistry, and engineering will need to advance transdisciplinary research cooperation with ecology, public health, and other disciplines.

### How can we ensure sustainable supplies of clean water, energy development, and food security while simultaneously minimizing ecological impacts and protecting environmental quality?

This question represents perhaps the grandest challenge of the 21st century. Increasing populations and levels of development across the globe are driving the need for sustainable supplies of clean water, energy development, and food security (UN 2015). In fact, the US National Academy of Science also identified the production of sustainable supplies of food, energy, and water as a grand challenge for environmental engineering in the 21st century (NASEM 2018). However, there is a need to ensure that any new technological advances to address a particular issue do not result in risk trade‐offs that have adverse impacts to environmental quality and ecosystem integrity. For example, sources of clean energy are being heavily promoted to mitigate climate change and poor air quality. In 2018 six solar panels were installed every minute in Australia, with 1 of every 5 households hosting rooftop solar generation (CER 2019). Over the next 10 y the use of solar technologies is expected to accelerate, and improved solar energy capture and storage materials are being developed. There is the potential for these materials to become sources for CECs and to enter waste streams as they are decommissioned and replaced. Therefore, as we move toward a circular economy, we must be mindful of the implications of new technologies for environmental quality. Better integration of robust predictive and comparative toxicology within life cycle assessment represents an important research opportunity.

## MULTIPLE STRESSORS AND MIXTURES

### What are the combined impacts of various agrochemicals (e.g., veterinary medicines, pesticides) and eutrophication from intensive terrestrial farming operations on the health of aquatic and terrestrial organisms?

Primary industry is a key economic driver in the Australasian region of Oceania. Intensive and industrial agricultural practices have resulted in increased levels of pollutants being discharged to the environment increasing the potential to impact associated ecosystems and adjacent landscapes. Agrochemicals and veterinary medicines often co‐occur in nutrient‐enriched ecosystems, yet ecotoxicology studies of these contaminants across nutrient gradients are rare (Brooks et al. 2008). Traditionally, ecological risk assessment of agrochemicals has been conducted on a chemical‐by‐chemical basis, but the cumulative effects of these chemicals with veterinary medicines, with other stressors (Gustavsson et al. 2017), or within eutrophic systems (Baxter et al. 2016) has not been robustly addressed. Common ecotoxicity assays with plants and algae often employ media with nutrient‐enriched concentrations and stoichiometric conditions that deviate from environmentally relevant conditions (Brooks et al. 2015). Further, nutrient‐enriched conditions can promote development of HABs and associated production of algal toxins, which are now recognized to confound stressor identification approaches for anthropogenic contaminants (Brooks et al. 2016).

More complex laboratory and (semi)controlled field studies are needed to assess the potential additive, antagonistic, or synergistic effects of these complex stressor mixtures. As one example, Taylor et al. (2018) recently demonstrated the usefulness of employing coupled field studies with experimental stream mesocosm experiments to identify ecological thresholds associated with P enrichment. Unfortunately, similar studies have rarely examined influences of agrochemicals or veterinary medicines, a number of which are actually pesticidal, on stream ecosystems across nutrient gradients. Aided by answering other priority research questions identified in the current paper, developing fundamental understanding of the specific Mode of Action (MOAs) of these chemicals will help determine their combined effects. However, the data generated need to be supported within ecological risk assessment models that are able to accurately predict cumulative effects, including ecosystems services (Syberg et al. 2017). Herein, future research at the intersections of ecological stoichiometry and toxicology (i.e., how nutrition can affect the toxicity of contaminants, how contaminants can influence nutrient dynamics, or how nutrients can influence toxins production) promises to support an understanding of interactive effects of anthropogenic contaminants and algal toxins in nutrient‐enriched systems (Conine and Frost 2016). Similarly, advances in ecological genomics are poised to support environmental assessment of complex stressors in the field (Yang et al. 2018; Zhang et al. 2018).

### What are the effects of changing demographics, economic development, consumption patterns, and climate (e.g., ocean acidity, water temperature) on chemical emissions, environmental fate, and ecotoxicology of contaminants and multiple stressors?

Anthropogenic stressors, including increased population, economic activity, and changing consumption patterns, are contributing to rapid environmental change (Steffen et al. 2015). The identified global megatrends of increased urbanization, diverging population trends, changing disease burdens, and accelerating technological growth will determine the types and quantities of chemicals released regionally (e.g., Kookana et al. 2014). Our current paradigms for environmental fate and toxicity of contaminants will be challenged by the anticipated increase in environmental pollution (EEA 2015) and the consequences of climate change. Global climate change is anticipated to alter both the environmental variables (e.g., temperature, precipitation, salinity, pH) that determine the environmental fate and toxicity of chemicals as well as the resilience of organisms to cope with exposure to chemical stressors (Hooper et al. 2013). Risk assessment tools and environmental surveillance systems will need to be sufficiently adaptive to identify and prioritize emerging threats, particularly those that arise due to a combination of chemical and physical stressors, some of which will be driven by global climate changes (Landis et al. 2013). Given the inherent difficulties in replicating “real world” conditions for experiments, our predictive modeling tools will need to be refined to ensure that a precautionary approach can be taken to managing risk in a rapidly changing world.

### What are the combined effects of very low levels of multiple contaminants (e.g., pesticides, natural resource extraction contaminants, salinity, pharmaceuticals and personal care products, endocrine‐disrupting chemicals) with different modes of action on aquatic and terrestrial organisms and ecosystems?

Understanding environmental consequences of chemical mixtures remains one of the most challenging issues in achieving sustainable environmental quality (Van den Brink et al. 2018; Fairbrother et al. 2019). With increasing urbanization, multiple land uses are interfacing in peri‐urban watersheds, which inherently increases the likelihood of diverse contaminants from urban, agricultural, and industrial activities that co‐occur in complex mixture scenarios. Guidelines derived for individual stressors may not be sufficiently protective when ecosystems are exposed to multiple stressors. For example, changes in benthic community distributions have been reported at concentrations below individual metal guideline values (Tremblay et al. 2017). Salinization is particularly relevant to regions in Australasia, yet influences of salinity gradients on contaminants with diverse modes of action are poorly understood among species (Canedo‐Arguelles et al. 2018). Various toxicity identification evaluation (TIE) protocols, response‐directed fractionation procedures, and effects‐directed analyses have been developed to identify causative chemical stressors within surface waters and sediments. However, it is particularly important to define strengths and limitations of historical bioassays employed for such activities, particularly when low levels of biologically active contaminants with diverse MIEs are considered. In recent years, bioassay tools with increasing mechanistic specificity have become important for diagnostic applications (Escher et al. 2014) beyond the traditional morphometric aquatic toxicity responses introduced above that are employed in TIEs (USEPA 1991). Unprecedented opportunities are emerging with use of high‐throughput in vitro, transgenic fish lines, and in situ toxicogenomic platforms when coupled with targeted and nontargeted chemical analyses (Bradley et al. 2017) in the field (Blackwell et al. 2017; Bradley et al. 2017; Perkins et al. 2017). However, metabolic transformation of contaminants and other basic scientific limitations remain when extrapolating in vitro to in vivo effects and even comparing responses among the 2 most common fish models (Corrales et al. 2016; Steele et al. 2018). Advancing AOP efforts for mixtures and predictive modeling of these complex low‐level constituents will be important. The funnel hypothesis (Warne and Hawker 1995) postulates that, as the numbers of chemicals present at equipotent concentrations increases, the likelihood of additive combined effects increases. Efforts are needed to identify whether, when, and what specific MOAs drive divergence from such theoretical constructs of low‐level mixture toxicity. It is thus not surprising that understanding the environmental implications of chemical mixtures was also identified as a priority research question in GHSP efforts from Europe (van den Brink et al. 2018), Latin America (Furley et al. 2018), and North America (Fairbrother et al. 2019). Clearly, this area deserves future attention.

## RISK ASSESSMENT, REGULATIONS, AND GUIDELINES

### What water quality guidelines are needed to protect temporary waters and associated ecosystems from the influences of development?

Temporary waters (i.e., intermittent, ephemeral, and seasonal) are common in temperate, arid, and semiarid landscapes of Australia and many other regions around the world. Sheldon and Fellows (2010) reported that up to 95 % of Australia's river channels are temporary, while a large proportion of the standing inland waters are also classified as temporary. Consequently, when these waters are present they are an extremely important source of water for the ecosystems of inland Australia and other regions. To date, much of the research has focused on the effects of extraction and sustainable use of temporary waters (Acuña et al. 2014; Datry et al. 2014), provision of their ecosystem services (Boulton 2014), and the importance of wetting and drying cycles for ecosystem health (Leigh 2013). However, there is a recognized need to better address changes in water quality arising from urbanization, agriculture, and mining (e.g., Queensland, Ramsay et al. 2012; South Australia, Botwe et al. 2015).

Due to the nature of these temporary waters, they are likely to experience pulse‐exposure scenarios, but there are limited data sets that are useful for determining water quality guideline values for episodic exposures to contaminants. Moreover, many temporary waters have been converted to perennial or near‐perennial waters by effluent discharges (Brooks et al. 2006), which represent important systems for environmental management with changing climatic conditions (Luthy et al. 2015). Although there are controls in Australia and some other countries on water quality in discharges and/or receiving waters for perennial or near‐perennial waters, no specific guidance exists in any set of guidelines or regulations on the combined impact of conversion from temporary to nontemporary status together with alteration of water quality. Understanding and managing environmental quality impairments in these temporary waters represents a timely research need for parts of the Australasian region of Oceania and other global systems experiencing urbanization and climate change.

### What are the effects of short magnitude, frequency, and duration (e.g., intermittent, episodic) exposures to contaminants and other stressors, and how can these scenarios be effectively incorporated into water quality guidelines?

Water quality criteria, standards, and guidelines are developed to protect various uses of surface waters. Through these efforts, threshold concentrations of contaminants (e.g., metals, pesticides, ammonia) and other stressors (e.g., depressed dissolved O, increased temperature) are identified and then applied, particularly in developed countries. Such regulatory “bright lines,” representing specific concentrations of individual contaminants, have historically been intended to be protective of, and ideally predictive of, ecological integrity. Presently, these numeric values are most commonly derived from probabilistic analyses of results from single‐species toxicity assays, which are intended to identify concentration–response thresholds, instead of individual species or community effects from episodic exposures that inherently vary in magnitude, frequency, and duration (Posthuma et al. 2001). For example, King et al. (2016) recently reported ecological structure and function responses to environmentally realistic episodic pulses of a common herbicide using outdoor stream mesocosms. Clearly, an advanced understanding of responses to episodic and intermittent chemical exposures is needed. Such information, while requiring innovative mechanistic coupling of toxicokinetics and toxicodynamics, and ecological genomics in the field, promises to reduce uncertainties associated with laboratory‐to‐field extrapolation during derivation of water quality guidelines.

### How can we measure ecosystem resilience to and recovery following exposure to stressors?

Stochastic events influence ecosystem services and biodiversity, which are among the most common protection goals identified during problem formulation of ecological risk assessments. Such stochasticity inherently affects interpretation of stressor‐response observations in the field and implementation of environmental management decisions. Although the diversity–stability hypothesis and functional redundancies have long been considered, both theoretically and empirically, and debated (McCann 2000) in ecology and ecotoxicology, identifying functional traits within assemblages and other ecosystem characteristics that impart resilience to natural and anthropogenic stressors remains decidedly challenging. In fact, 2017 has been described as the year of the disaster, with numerous billion‐dollar events reported throughout the world (NOAA 2018). Herein, ecosystem services, when not compromised, represent key management objectives for disaster risk reduction and climate change adaptation (Monty et al. 2016; Renaud et al. 2016), and are appropriately included in the United Nation's Sendai Framework for Disaster Risk Reduction for 2015 to 2030 (UNDRR 2015). For example, rapid global declines of terrestrial and aquatic species present a profound manifestation of cumulative threats to biodiversity. In Australasia, degradation of the Great Barrier Reef has prompted extensive efforts to define cumulative stressors and advance resilience‐based management (Anthony et al. 2013). In New Zealand, large earthquakes in the Canterbury region resulted in loss of habitat and measurable stress on aquatic organisms (Potter et al. 2015; Chandurvelan et al. 2016). Similarly, the Rena oil spill, New Zealand's largest maritime environmental disaster, impacted hundreds of kilometers of coastline in 2011 (Schiel et al. 2016). In such cases, influences of rare species on ecosystems functions require additional study (Leitao et al. 2016). With the prospects of climate change further compounding multiple stressor effects on aquatic and terrestrial ecosystems, it appears clear that developing an advanced understanding of ecosystem resiliency prior to and following disasters and in the face of cumulative stressors has never been more important.

## SPOTLIGHT ON AUSTRALASIA

### Are there differences in toxicological thresholds among native and nonnative organisms, and how can species sensitivity information from nonresident species be used to predict adverse outcomes and protect our unique biota and ecosystems?

The iconic aquatic and terrestrial species unique to Oceania in general and Australasia in particular hold deep cultural significance to indigenous communities and are important to the recreational, commercial, and conservation sectors. However, most of the toxicity estimates are derived from studies that use North American and European species; very little toxicity data exist using Oceania species, with some notable exceptions. Consequently, the Australian and New Zealand Water Quality Management Strategy (ANZECC 2000), and the new revised guidelines took the pragmatic approach of deriving Default Water Quality Guideline Values using any available data that passed predefined quality control criteria. However, this approach makes the considerable assumption that native Oceania species are of a similar sensitivity to that of nonnative species. This assumption has not been comprehensively tested because there have been no broad‐scale systematic comparisons on toxicity data from native Oceania species and nonnative species. It is important to note that a similar question was recently identified from Latin America (Furley et al. 2018). Advancing comparative and predictive toxicology research promises to help us understand differences among species sensitivities to contaminants with diverse mechanisms of action (Brooks 2018).

There have been many toxicity tests developed for native species in Australasia. The earliest of these native‐species suites were developed to satisfy the research needs for controversial issues. For example, in the early 1990s, the National Pulp Mills Research Programme identified a number of temperate Australian species to assess the toxicity of pulp mill effluents and test “greener” technology options (Crossland and Abel 1992; Stauber et al. 1994). In New Zealand, a standard suite of 3 marine and 4 freshwater tests on native species was developed by the National Institute of Water and Atmospheric Research (Hall and Golding 1998), and sensitivities of these species were compared with those of nonnative species for 4 reference toxicants. A suite of standardized tropical freshwater toxicity tests was developed by the Environmental Research Institute of the Supervising Scientist for the regulation of the Ranger Uranium Mine, which is adjacent to the World Heritage–listed Kakadu National Park (Riethmuller et al. 2003). Both of these industries were faced with significant public opposition, but the development of native‐species toxicity tests helped decision makers reassure the public that environmental issues were being addressed appropriately.

In more recent years, a member of the business community invested in the development of a suite of toxicity tests using native tropical marine species to improve the environmental management of their industrial effluents by using biological effects data (van Dam et al. 2018). The motivation for this was to address a gap that existed for tropical species because most toxicity tests were developed by first‐world nations in temperate environments (van Dam et al. 2008). Such research investments have subsequently benefited other industries that have capitalized on the availability of the tropical tests (e.g., Gissi et al. 2018), which has enabled valuable tropical‐versus‐temperate comparisons (Peters et al. 2019). Ad hoc toxicity testing using culturally significant fishes (e.g., Inanga, *Galaxis maculatus;* McRae et al. 2018) and invertebrates (e.g., freshwater mussels, clams, and crayfish; Clearwater et al. 2014) has been developed in New Zealand and Australia (e.g., Markich and Camilleri 1997). The sensitivities of native and nonnative species to certain contaminants have been compared in some cases. For example, Hagen and Douglas (2014) asked this question but could find sufficient data for only 3 chemicals, that is, 4‐chlorophenol, phenol, and ammonia. They concluded that there were no differences in species sensitivity that warranted the application of safety factors. However, until a sufficient Oceania data set for a broader set of chemicals is available, this question will remain unaddressed. Here again, advancing comparative ecotoxicology research in this area is a priority.

### How do we incorporate and protect cultural and social values (relating to humans, biota, and ecosystems) to empower citizen, societal, and indigenous engagement in the research, management, and legislation of priority environmental contaminants?

Indigenous peoples are key to many environmental management projects and decisions globally, where their status ranges from disadvantaged minorities to the dominant cultural group within their respective communities and country. Indigenous peoples carry with them distinctive and localized cultural and environmental knowledge, based on thousands of years’ experience (Stevenson 1996). However, mechanisms to incorporate their indigenous knowledge, cultural values, and traditional management systems into decision‐making processes remain poorly formulated in most global legislatures, business decisions, and academic programs. This is the case despite numerous international and regional, legally and nonlegally binding instruments (Convention on Biological Diversity 1992; UN 1992, 2007) and statutory national obligations (legislative and policy level; Palmer 2008) requiring appropriate and meaningful indigenous peoples’ involvement. Further, ignorance of inherent challenges around the application of indigenous knowledge, existing power relations, and contextual nuances of Indigenous knowledge have also hampered access to, and an articulation of indigenous knowledge in, environmental management and decision‐making processes (Briggs 2005).

Oceania, like other global regions, has a diverse range of indigenous peoples, each with their own unique history, experiences, and challenges with respect to articulating their voice around environmental contaminants. Unfortunately, indigenous knowledge and values (IK&V) are not well represented in assessment and management approaches in environmental issues. Applying an indigenous knowledge lens considers the whole of environmental change in determining the impact of contaminants (Kookana et al. 2013). In addition to considering the impact of contaminants to indigenous people's environments, biodiversity, and culture (Ataria et al. 2016), the impact of practices that disrupt ecological patterns and services are also critical to consider, particularly for those communities that are reliant on natural resources for their physical and cultural existence.

The collaboration of traditional knowledge and research is needed between communities and indigenous peoples. Advancing forward it will be imperative to manage environmental quality as both strive to advance their knowledge systems to protect environmental quality and natural resources. Engagement protocols differ across all indigenous peoples globally. However, the environmental science and engineering communities can assist in cocreating protocols in close consultation with the relevant indigenous peoples that are specific to regions, are equitable, empower mutual benefit, and are enduring. Indigenous people assert an inherent expectation to be involved in caring for, protecting, and rejuvenating their traditional land, freshwater, marine, and atmospheric environments. To some it is a cultural obligation as custodians, whereas to others it is a means of maintaining their identity by reinstating and retaining their cultural practice and heritage and by empowering their developmental aspirations for future generations. Here we call for concerted global research efforts to integrate IK&V during problem formulation and, more specifically, identification of ecosystem protection goals within environmental risk assessment and management efforts.

## TOOLS FOR IMPROVING RISK ASSESSMENT

### How do we exploit, collate, and integrate existing environmental toxicology, chemistry, and geospatial data to help develop robust risk assessment?

Natural ecosystems are increasingly degraded as a result of exposure to multiple stressors that vary over space and time. We now know that the global reach of anthropogenic stressors is beyond what was previously predicted, with persistent pollutants such as PCBs, polybrominated diphenyl ethers (PBDEs), and microplastics found in the remote Arctic and deep sea trenches (Schlining et al. 2013; Van Cauwenberghe et al. 2013; Obbard et al. 2014; Jamieson et al. 2017). To address these challenges, we have increasing access to physical, biological, and chemical measurements from new remote sensing tools and their integration into geographical information systems (Dafforn et al. 2016). Moreover, advances in molecular analysis have allowed us to capture more holistic information about the health of entire ecosystems, from microbial to macrobiotic scales, and to go beyond impacts on structure to understand consequences for ecosystem function and services (Chariton et al. 2016). The advent of real‐time technologies such as the MinION for DNA/RNA sequencing and the microfluidic lab‐on‐a‐chip provides us with more opportunities for improved spatiotemporal analyses (Campana and Wlodkowic 2018). The availability of these data and new geospatial and ecogenomic bioassessment tools has the potential to increase our capacity for ranking and understanding stressor impacts and crucially to allow us to differentiate stressors impacts when present in combination.

At the same time, we are experiencing technological advances and associated information booms, with many decades of ecotoxicological testing and biomonitoring information collected and added to databases following regulatory requirements. Numerous databases around the world hold information about different chemical stressors as well as potential biological responses. For example, the Pesticide Properties DataBase has approximately 2300 pesticide active substances and >700 metabolites stored alongside response metrics related to human and environmental health (Lewis et al. 2016). Other large collections of biological data such as GENBANK (Benson et al. 2010), TRY (Kattge et al. 2011), D3 (Hintze et al. 2013), COMADRE, and COMPADRE (Salguero‐Gómez et al. 2015) offer information related to genetics, functional plant ecology, grassland ecology, and plant and animal demography alongside metadata from, for example, ecoregions that can be used to ask globally relevant questions (Salguero‐Gómez et al. 2015) and be integrated within risk assessment frameworks.

Machine learning techniques could be used to harness the power of such extensive data sets into risk assessment. For example, molecular tools such as transcriptomics have been integrated with machine learning techniques to identify and classify priority EDCs (Ornostay et al. 2013). Similarly, artificial neural networks have been used to select biomarkers on the basis of key response variables (Bradley 2012). Decision tree models based on environmental metadata have been used to predict benthic macroinvertebrate distributions (D'Heygere et al. 2003). Environmental metadata using a Random Forests machine learning algorithm have likewise been used to reveal nonlinear relationships and critical thresholds for cyanobacterial blooms (Nelson et al. 2018), which is significant because HABs now represent the greatest water quality threat in some ecosystems (Brooks et al. 2017).

Overall, our predictive power has exponentially increased, allowing us to move beyond the current norm of single‐stressor assessments, done at small spatial scales and with few receptors, to enhanced risk assessment (Van den Brink et al. 2016). However, there are still hurdles to overcome before we can harness and exploit this Big Data to its fullest. We need to 1) improve our techniques for data validation to remove errors in, for example, specimen identifications for DNA barcoding; 2) improve the availability of data not just through openness but also by targeting underrepresented taxonomic and geographic groupings; 3) improve standardization so that data are comparable over space and time; and 4) invest in real‐time technologies that provide direct measures of impact rather than providing proxies (Dafforn et al. 2016).

### How can prescreening techniques (e.g., in silico, in vitro) be developed, advanced, and validated to identify and predict whole organism effects?

The rate of discovery and synthesis of new chemicals has grown exponentially in the last decades, exceeding our ability to empirically determine the toxicity of new compounds using conventional (whole animal) toxicity testing methods. This means that more and more chemicals are put into global circulation without a thorough understanding of their potential toxic impacts. Too often, the chemicals substituted for problematic substances display unacceptable toxicity profiles (Rosal et al. 2010; Björnsdotter et al. 2017). Unfortunately, conventional toxicity testing provides too narrow a funnel (in terms of time, cost, and ultimately, throughput) to assess the risk of the vast number of new compounds designed daily by chemical, pharmaceutical, and agricultural industries. Clearly, a higher throughput approach is required.

This is where in silico modeling and in vitro pretesting methods offer a way forward. Using these HTS techniques, which can screen thousands of chemicals every day, toxicity testing can be prioritized and focused on those molecules most likely to pose a threat to humans and/or ecosystems (Collins et al. 2008). This is the paradigm shift foreshadowed in the Tox21 vision for toxicity testing in the 21st century (NRC 2007), and which relies on the AOP concept (Ankley et al. 2010) to translate a key initiating event at the molecular or cellular level (either modeled in silico or measured in vitro) to the adverse outcome of consequence (e.g., survival, reproduction, development, behavior) that is our focus of concern (Ankley et al. 2016).

Although tremendous progress has been achieved in adapting and validating in vitro tools to environmental monitoring and risk assessment (e.g., in Australasia, Coleman et al. 2008; Mispagel et al. 2009; Chinathamby et al. 2013; Bain et al. 2014; Escher et al. 2014; Leusch et al. 2014; Scott et al. 2014; Roberts et al. 2015; Boehler et al. 2017; Neale, Achard et al. 2017; Neale, Altenburger et al. 2017; Chen et al. 2018; Leusch et al. 2018), some fundamental questions still need to be systematically addressed before these techniques can become reliable predictors of whole animal level effects:

1) Refine quantitative in vitro to in vivo extrapolation (QIVIVE): Although there is a clear correlation between in vitro response and in vivo effects for some endpoints such as acute toxicity (Kaiser 1998; Tanneberger et al. 2013; Natsch et al. 2018) and receptor‐mediated endocrine effects (Sonneveld et al. 2006, 2011; Henneberg et al. 2014), toxicokinetic factors (absorption, distribution, metabolism, and excretion) still pose a difficult challenge for QIVIVE (Blaauboer 2015; Meek and Lipscomb 2015), although groundbreaking studies suggest that this may soon be within reach (Rotroff et al. 2010; Wetmore 2015).
2) Fully map relevant AOPs: There is still much work to be done to map key events (KEs) to connect the dots between the molecular or cellular initiating event and the ultimate apical consequence to produce comprehensive AOPs, for both humans and ecosystems (Ankley et al. 2016). In combination with QIVIVE, this mapping would ultimately allow us to produce quantitative AOPs.
3) How much is too much? In vitro assays are often exquisitely sensitive and able to detect activity even in clean samples. In whole organisms, a small amount of dysfunction at the molecular and cellular level can often be compensated for by defense and repair mechanisms to avoid any higher level consequence. Until we can quantitatively extrapolate from in vitro to in vivo (steps 1 and 2 above) and quantify the repair ability for each type of dysfunction, it will be difficult to accurately link an in vitro response to an in vivo adverse effect. In the meantime, several different approaches have been proposed to produce effects‐based trigger (EBT) values, including reading across from current chemical guidelines (Escher et al. 2015, 2018) or de novo derivation (Brand et al. 2013; Jarošová et al. 2014) and how to use them in a practical context (Leusch and Snyder 2015; Ron et al. 2017).

Clearly, there are still some unanswered questions in how we use in silico models and in vitro bioassays. But these new tools also offer a unique and necessary solution to overhaul the single‐chemical risk assessment approach that relies on the traditional aquatic models and endpoints discussed above and to properly screen the sheer number of chemicals that make our modern lifestyles possible without negatively impacting human health and the environment. Further, advancing these diagnostic tools, particularly when coupled with nontarget analytical methods, promises to support efforts to answer other priority research questions identified here.

### How can ecotoxicology information be integrated more closely during interpretation of ecological data?

Two closely related questions focus on the necessity of more closely integrating research among ecology and ecotoxicology, which in many parts of the world remain separate fields of study. Whereas basic ecology studies in terrestrial and aquatic systems are fundamentally important for conservation, including understanding ecosystem services and biodiversity, translational ecological efforts remain critical for environmental assessment and management (Saaristo et al. 2018). Interpretation of field data sets can be challenging due to ecosystems commonly being exposed to multiple stressors, which may be known or unknown. Subsequently, identifying underlying causative relationships among complex stressors requires multidisciplinary perspectives. For example, failure to consider chemical stressors beyond nutrient enrichment during basic ecological and biogeochemical studies in systems influenced by agriculture and urbanization can confound interpretation of findings. For decades, researchers have called for close integration among ecology and ecotoxicology research pursuits (Cairns 1988; Zala and Penn 2004; Melvin and Wilson 2013; Arnold et al. 2014).

More recent contributions in community and stream ecology (Rohr et al. 2006; Rosi‐Marshall and Royer 2012; Bernhardt et al. 2017), behavioral ecology (Saaristo et al. 2018), and ecophysiology (Cooke et al. 2013) consistently echo these earlier sentiments. Beyond applied studies aimed at stressor identification, anthropogenic chemicals, particularly specifically acting contaminants (e.g., pesticides, pharmaceuticals), can serve as experimental scalpels to dissect basic structural and functional relationships. For example, mesocosm studies by Fairchild et al. (1994) with pesticides partitioned direct from indirect community interactions. Environmental studies with pharmaceuticals have yielded unique comparative ecophysiology information (Owen et al. 2007). Addressing several of the questions identified in earlier sections aimed at advancing integrated research in ecological threshold analyses, environmental genomics, quantitative AOPs, and integrative, comparative, and predictive toxicology, when coupled within mainstream experimental and theoretical ecology, promises reciprocal and transformational basic and applied benefit, particularly as global ecosystems continue to be influenced by complex stressors.

### How do we advance ecotoxicology testing to be more relevant to ecological systems?

Prospective ecotoxicology assays are employed by businesses and government agencies to assess the safety of substances prior to their introduction to the market or to assess contaminants of potential concern before they are released to the environment. Industrial operations have also been required to synthesize predicted effluents for safety assessments when changing their waste treatment or introducing new ones. Historical products in commerce may also be prioritized for more detailed safety assessment. Whereas retrospective ecotoxicological studies often include in vitro and in vivo models to examine field‐collected water, sediment, or soil in laboratory settings, in situ studies with caged organisms, and surveillance of biological conditions in the field, micro‐ and mesocosm studies are employed for both prospective and retrospective efforts in an attempt to bridge laboratory‐to‐field information. For decades, researchers have noted challenges from lower to higher scales of biological complexity due to increasing endpoint variability (and societal relevance) and environmental stochasticity as one moves from the laboratory model to ecosystem‐level perturbations (Dickson et al. 1992; La Point and Waller 2000).

Predictive coupling of laboratory with field perturbations remains a grand challenge in environmental science. However, it remains important to ensure the quality of data produced from standardized model systems, while advancing innovative and exploratory ecotoxicological research that may not be intended or amenable to directly be integrated within environmental assessments (Moermond et al. 2017). Such challenges were considered during a recent SETAC Pellston Workshop on “Improving Usability of Ecotoxicology in Regulatory Decision Making, August 2015” which has documented the need for ecotoxicological data sets that are reliable and relevant (Rudén et al. 2017). Beyond the traditional biological indices approaches, recent progress in ecological threshold analysis (Baker and King 2010), ecological genomics (Zhang et al. 2018), and species traits (Van den Brink et al. 2013) are improving field studies. Future research in mechanistic and comparative ecotoxicology, if integrated with ecology, is poised to support more robust experimental designs and extrapolations across levels of biological organization, although uptake of recent advances within prospective and retrospective regulatory activities remains differential around the world. Therefore, employing reasonable and defensible weight‐of‐evidence approaches will remain important (Suter 2006).

## CONCLUSIONS

The Australasian region of Oceania faces increasingly diverse environmental challenges associated with multiple stressor influences on environmental quality. The current analysis represents an initial attempt within Oceania to develop a research agenda aimed at advancing toward more sustainable environmental quality and ecosystem integrity. Through a transparent, bottom‐up, multidisciplinary, and multistakeholder process, we identified 20 priority questions to support future environmental research. As noted recently (Van den Brink et al. 2018), step changes are needed for basic and applied studies of environmental stressors, and their management, if we are to achieve the United Nation's Sustainable Development Goals (UN 2015). We agree, as evidenced by the interconnections among priority research questions reported herein.

Several questions identified the need to improve predictive environmental exposure and toxicology tools for risk assessment and to reduce and replace animal testing. Similarly, the development of robust nontarget analytical screening techniques to determine priority contaminants in ecosystems exposed to complex mixtures was identified as an urgent need. Strategically advancing these areas will assist in addressing other questions related to multiple stressors (e.g., chemicals, salinity, acidification), susceptibility of regional flora and fauna, management of unique ecosystems (e.g., ephemeral water bodies), and stress from global megatrends (e.g., urbanization, the food–energy–water nexus) and climate change. The importance of understanding the comparative sensitivities of regionally unique species was also reported from Latin America (Furley et al. 2018). Incorporating and protecting cultural and social values to empower citizens, especially indigenous peoples’ engagement during research, management, and policy development, was further identified as a key research opportunity. In this regard, ongoing efforts within Australasia are incorporating cultural knowledge during identification of ecosystems protection goals (i.e., the Whanganui River and other systems in New Zealand have been granted the same legal rights as a person), which represents an interesting model that could benefit elsewhere.

We expect the top 20 questions identified here will be complementary to and assist advancement of national prioritization efforts such as the Australian Science and Research Priorities and Practical Challenges (Australian Government 2015) and the New Zealand National Science Challenges (MBIE 2016). For example, 5 of the 11 Australian Science Research Priorities (e.g., Environmental Change, Energy, Soil, Water, Food) include Practical Challenges to address sustainable environmental quality and ecosystem integrity. Similarly in New Zealand, Science Challenges relevant to sustainable environmental quality include Biological Heritage, The Deep South, Sustainable Seas, and Our Land and Water. Expertise and capacity within the Australasia chapter of SETAC and other scientific disciplines in Oceania are well positioned to support these efforts (a brief history of SETAC Australasia can be found in the Supplemental Data). Answering the 20 priority research questions will not be trivial, but will support basic and applied research innovation and advancement of robust practices to achieve more sustainable environmental quality within the region and other parts of the world.

## Disclaimer

The authors declare no conﬂicts of interest. The peer‐review process for this article was managed by the Editorial Board without the involvement of S Gaw.

## SUPPLEMENTAL DATA

Background information and the full set of questions submitted:

1) A Brief History of the SETAC Australasia Chapter
2) Author‐submitted Questions

## Supporting information

This article contains online‐only Supplemental Data.

Supporting information.

Supporting information.
