# Supplementary material for: Towards Sustainable Environmental Quality: Priority Research Questions for the Australasian Region of Oceania
Source: Integr Environ Assess Manag. 2019 Sep 13;15(6):917–35. doi: 10.1002/ieam.4180 (PMC6899907; doi:10.1002/ieam.4180)
Supplement: Supplementary file 2 — Supporting information. [file IEAM-15-917-s002.docx]

**A Brief History of the SETAC Australasia Chapter**

The forerunner of SETAC-AU chapter was the Australasian Society for Ecotoxicology (ASE), which commenced at its inaugural conference in Sydney in 1994. The ASE brought together the scientists representing developing activity in ecotoxicology in the region through the 1970s and 1980s. Apart from ecotoxicological monitoring of the Ranger Uranium Mine in Kakadu National Park by the Australian Office of the Supervising Scientist facilities (now ERISS), there was, in the early 1980s, little legislative backing for ecotoxicology testing in Australia. Nevertheless, government, university and industry laboratories throughout Australia were developing acute and chronic tests with locally relevant species. The New Zealand Resource Management Act (1991) (RMA; Hickey 1995a) gave impetus to development of locally relevant freshwater and marine tests, with an emphasis on sensitivity of native species, detection of effects at low levels of contamination and addressing the requirements of the RMA that discharges to receiving waters do not cause “adverse effects” (Hickey 1995b). The establishment of ASE, which brought together these disparate groups, helped to accelerate development of regional methods for freshwater and marine systems, including mesocosm and field studies, sediment and soil ecotoxicology and increased understanding of the sensitivity of native species and how physiological, and climatic parameters affect toxicity. One tangible product of this was the publication of databases of Australasian ecotoxicology data for metals (Markich et al 2002), organic chemicals (Warne et al 1999), pesticides (Warne et al 1998) and contaminants in soil (Langdon et al 2009). The ASE was involved in the development of the Australian and New Zealand Guidelines for Fresh and Marine Water Quality (ANZECC and ARMCANZ 2000) for around 200 chemicals; these guidelines use, where possible, probabilistic approaches for extrapolation of ecotoxicology data. In 2008, ASE’s “Ultimo Declaration” advocated the need for revision of the 2000 Guidelines, which were recently completed. The ASE provided an Expert Reference Panel, which developed methods for the current revision at an early stage and assisted with peer-review, while many members were involved in deriving Guideline figures and fact-sheets.

The ASE was involved in the developing field of micropollutants including endocrine disrupting chemicals (EDCs) and pharmaceuticals and personal care products. These activities led to a continuing series of “What’s in Our Waters” conferences and fostering of international collaborations with the UK and Japan and development of an Australian national position paper produced in November 2007 as “The Black Mountain Declaration on EDCs in Australian Water”. This document summarises the broad areas of consensus among a group of Australian and International researchers, policy makers, regulators, water suppliers and research investors on the state of knowledge about EDCs and the priority areas for future research, policy attention and public awareness ( http://lwa.gov.au/products/pn21411).

Since 1994, ASE, and now as SETAC-AU, have been filling knowledge gaps in such areas as method development, sensitivity of native species and developing regional test species, addressing environmental parameters that affect toxicity, developing environmental guidelines for water, sediment and soil, developing robust biomonitors, bioaccumulation, endocrine disruption and new and emerging chemicals.

**REFERENCES**

ANZECC and ARMCANZ. 2000. *Australian and New Zealand guidelines for fresh and marine water quality*. Australian and New Zealand Environment and Conservation Council and Agriculture and Resource Management Council of Australia and New Zealand, Canberra, ACT, Australia.

Hickey CW. 1995a. Legislation and ecotoxicity testing. *Australasian Journal of Ecotoxicology*, 1: 83-84.

Hickey CW. 1995b. Ecotoxicity in New Zealand. *Australasian Journal of Ecotoxicology*, 1: 43-50.

Langdon K, Warne M St J and Sunderam R I M. 2009. A compilation of data on the toxicity of chemicals to species in Australasia. Part 4: Metals (2000-2009)*. Australasian Journal of Ecotoxicology*, **15**: 51-184.

Markich SJ, Warne MStJ, Westbury A-M and Roberts CJ. 2002. A compilation of data on the toxicity of chemicals to species in Australasia – Part 3: Metals*. Australasian J. Ecotoxicol.* 8: 1-138.
